# Methadone-induced Torsades de Pointes Masquerading as Seizures

**DOI:** 10.5811/cpcem.2016.11.32664

**Published:** 2017-01-17

**Authors:** David C. Traficante, Gordon Feibish, John Kashani

**Affiliations:** * St. Joseph’s Regional Medical Center, Department of Emergency Medicine, Paterson, New Jersey; † Saint Vincent Hospital, Department of Emergency Medicine, Worcester, Massachusetts

## Abstract

The authors herein present the case of a 53-year-old female who was being treated as an outpatient for seizure disorder but was also receiving high-dose methadone therapy. She presented to the emergency department (ED) for what appeared to be a seizure and was found to have a prolonged QT interval, as well as runs of paroxysmal polymorphic ventricular tachycardia with seizure-like activity occurring during the arrhythmia. The markedly prolonged QT interval corrected after treatment with intravenous magnesium; subsequent electroencephalogram, neurology and cardiology consultations confirmed the cause of the recurrent seizure-like episodes to be secondary to the cardiotoxic effects of methadone.

## INTRODUCTION

QT interval prolongation and associated torsades de pointes (TdP) are well-documented side effects of methadone. Moreover, case reports illustrating the development of TdP in patients using methadone appear to be associated with higher doses of the medication.^1^ TdP is usually a self-remitting non-perfusing arrhythmia commonly manifesting as syncope or seizures. This case highlights the need to consider arrhythmias in the differential of a patient with seizures and/or presumed seizure disorder and emphasizes the importance of obtaining an electrocardiogram (EKG) in such patients.

## CASE REPORT

A 53-year-old Caucasian female presented to the emergency department (ED) after having what appeared to be a single seizure just prior to arrival, witnessed by her husband. He described generalized shaking lasting approximately 10 seconds with an associated brief period of loss of consciousness but no reported gaze defect, bowel or bladder incontinence, apnea or tongue biting. There was a 10–15 minute period of decreased responsiveness and confusion that occurred after the event. The patient’s husband reported she was evaluated at another ED two weeks prior for a similar episode. During that visit her labs and computed tomography (CT) of the head were negative. The patient refused admission at that time because she was concerned that she would not receive her methadone. Subsequently, she was given a prescription for levetiracetam and followed up with a neurologist the next day.

On the day of her presentation to our ED she was on her way to a scheduled EEG and magnetic resonance imaging of her brain. The husband reported that the patient had been having one to two seizures daily for the preceding two weeks, despite taking the prescribed dose of levetiracetam. The patient stated that she did not recall having the seizures; however, she described feeling palpitations and shortness of breath just prior to developing these episodes. There was no prior history of seizure disorder or recent head trauma. Her past medical history was significant for anxiety, for which she took alprazolam 0.5 mg twice daily, and heroin dependence for which she took methadone 140 mg daily. Emergency medical services reported her blood sugar to be 125 mg/dl and she appeared post-ictal with mentation that gradually improved during transport. On arrival to the ED, the patient was awake, well developed and well groomed, but was slow to respond. Her vital signs were blood pressure 129/81 mmHg, pulse 62 beats per minute (bpm), respiratory rate of 17 breaths per minute, temperature of 97.3 degrees Fahrenheit, and pulse oximetry of 100% on room air. There were no obvious signs of head trauma and pupils were round, 3 mm bilaterally and reactive to light with extra-ocular muscles intact. Cardiac, lung and abdominal exam were unremarkable. The patient was alert and oriented to person, place, time and situation. Her Glasgow Coma Scale was 15 and she had no focal neurological deficits.

**Image 1:**
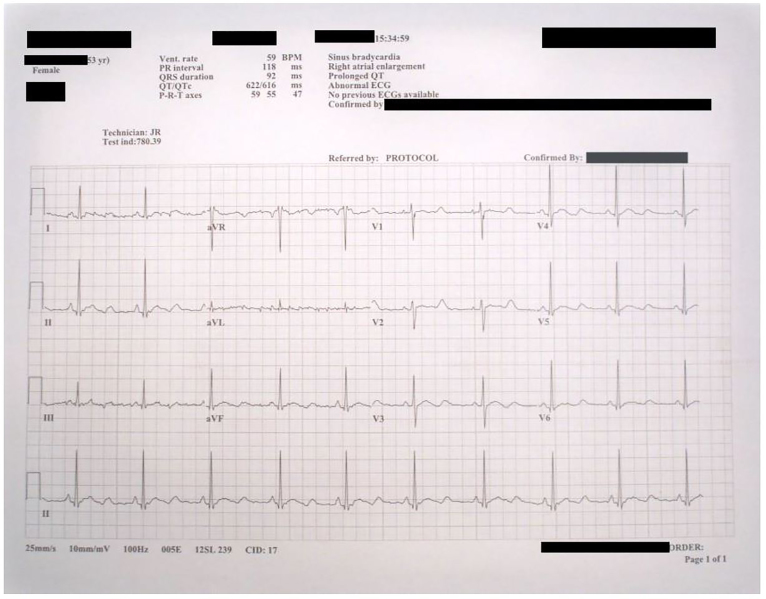
Initial electrocardiogram on patient arrival to the emergency department.

**Image 2:**
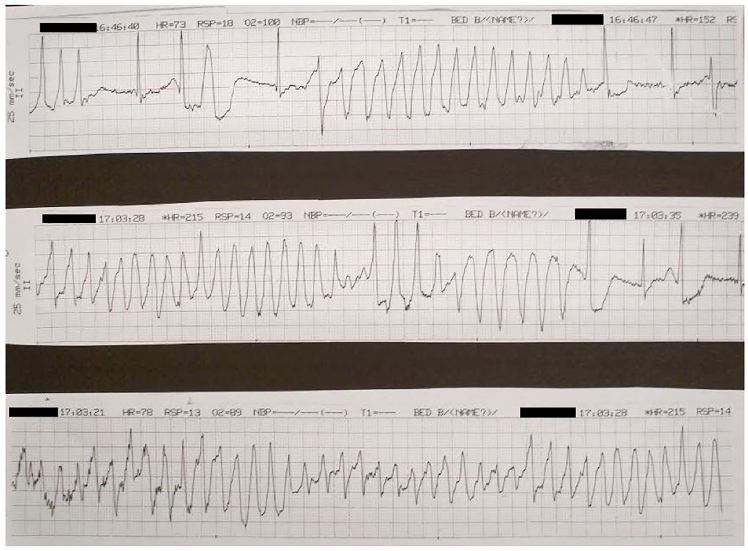
Rhythm strip demonstrating runs of Torsades de Pointes.

On arrival to the ED the patient was placed on a cardiac monitor and an EKG was obtained (Image 1). The EKG showed sinus bradycardia at a rate of 59 bpm with normal axis but the QT was prolonged at a rate of 622 msec. The patient was immediately given magnesium 2 grams intravenous and laboratory exams were obtained, which returned unremarkable. While in the ED the patient was noted to have multiple premature ventricular complexes on the telemetry monitor, which developed into two separate short runs of non-sustained paroxysmal polymorphic ventricular tachycardia (Image 2). During the first of these episodes the patient reported feeling palpitations and shortness of breath. During the second, longer event, the patient was witnessed to have an abbreviated episode of generalized tremor, which the husband reported to be similar to her prior occurrences of seizures. She also appeared post-ictal after this episode for approximately five minutes. Her blood pressure remained stable throughout the entire course. In addition, the patient received additional intravenous infusions of magnesium during and after these episodes. A CT of the head was obtained in the ED and was unremarkable for any acute pathology. A repeat EKG was obtained prior to patient transfer to the cardiac care unit, and after the magnesium infusions, which showed shortening of the QT length to 363 msec.

During her hospitalization neurology and cardiology consultations were obtained. An electroencephalogram was unremarkable for evidence of seizure activity and the cardiologist and neurologist collectively agreed that the prolonged QT and development of non-sustained polymorphic ventricular tachycardia were likely secondary to the high dose of methadone and the cause of the perceived seizure disorder. The patient’s QT interval remained within normal limits after receiving the doses of magnesium in the ED and throughout the remainder of her admission to the hospital. There was no recurrent seizure-like activity or arrhythmias during her hospitalization. She declined the placement of a defibrillator, which was recommended by cardiology since she refused to decrease her methadone dose. Both the physician who prescribed the patient’s methadone and her primary care physician were notified about her hospital course and workup. Despite her reluctance, the patient was tapered to a lower dose of methadone and was subsequently followed closely by her primary care physician for monitoring of her EKG. In addition, she was prescribed magnesium oxide 400 mg tablets twice daily prior to her discharge.

## DISCUSSION

Methadone is a long-acting synthetic opioid that can be used for pain control; however, its main use is as a maintenance program in patients with a history of heroin abuse.^2^ It has several known side effects, most importantly respiratory depression and cardiac toxicity. Specifically, methadone has been shown to increase QT intervals and is associated with the development of TdP. The development of QT prolongation by methadone is secondary to blockade of the delayed rectifier potassium current through the cardiac human ether-a-go-go related gene (hERG) channels.^3^ This occurs in a dose-dependent fashion putting those on chronic therapy with higher doses to be at risk for QT prolongation.^4^ The incidence of some degree of QT prolongation has been reported in more than 80% of patients on methadone maintenance therapy. More profound QT prolongation (>500 msec) in this population, however, has been reported in the literature to have an incidence of 2.4% to 16.6%.^5^,^6^ The exact incidence of methadone-induced TdP is unknown; however, separate studies report an incidence of between 0.3% to 3.6%.^7^,^8^ TdP is a specific form of polymorphic ventricular tachycardia that is commonly associated with patients with prolonged QT interval. Patients with TdP may present with sudden cardiac arrest, but more typically present with recurrent episodes of dizziness, palpitations, syncope or what may be perceived as seizure.^9^–^11^ The reporting of seizure occurring during an episode of TdP has been reported in the medical literature.^12^–^15^ Our patient was presumed to have a recently diagnosed seizure disorder and was started on antiepileptic medication; this differs from previously reported cases in which the patients were not being treated for seizure disorder as outpatients. The witnessing of what appeared to be a seizure occurring during an episode of TdP in our patient, as well as the negative work-up for seizures, solidifies the diagnosis of TdP-induced central nervous system hypoperfusion appearing to be a seizure. Although our patient was also on alprazolam, the potential for alprazolam withdrawal as the etiology of her seizures is unlikely as she was reportedly compliant with her medications. In addition, apart from alprazolam and levetiracetam, which may have mildly contributed to the patient’s QT interval, she was not taking any other medications that could have prolonged the QT interval.

## CONCLUSION

The authors believe the patient’s manifestation of seizure episodes was secondary to the cardiotoxic effects of her high-dose methadone treatment. After receiving intravenous magnesium in the ED, with subsequent improvement in the QT interval and resolution of further episodes of TdP, the patient had no further episodes of seizure activity. More likely there were no epileptic seizures; instead, epileptiform motor activity was secondary to cerebral hypoperfusion due to runs of paroxysmal polymorphic ventricular tachycardia. This case serves as an important reminder for why EKGs are vital diagnostic tools for a patient presenting with seizures, especially for those patients taking methadone or other drugs that are associated with QT interval prolongation. Healthcare practitioners should consider obtaining an EKG in patients presenting with seizures and should inquire about the use of methadone and other drugs that can predispose a patient to arrhythmias.
